# Experimental Pore-Scale Study of a Novel Functionalized Iron-Carbon Nanohybrid for Enhanced Oil Recovery (EOR)

**DOI:** 10.3390/nano12010103

**Published:** 2021-12-29

**Authors:** Fatemeh Razavirad, Abbas Shahrabadi, Parham Babakhani Dehkordi, Alimorad Rashidi

**Affiliations:** Research Institute of Petroleum Industry, Tehran 1485613111, Iran; shahrabadia@ripi.ir (A.S.); babakhani65@gmail.com (P.B.D.); rashidiam@ripi.ir (A.R.)

**Keywords:** iron-carbon nanohybrid, oil-wet micromodel, enhanced oil recovery, wettability, alteration, heavy oil

## Abstract

Nanofluid flooding, as a new technique to enhance oil recovery, has recently aroused much attention. The current study considers the performance of a novel iron-carbon nanohybrid to EOR. Carbon nanoparticles was synthesized via the hydrothermal method with citric acid and hybridize with iron ($Fe_{3}O_{4}$). The investigated nanohybrid is characterized by its rheological properties (viscosity), X-ray diffraction (XRD), and Fourier transform infrared spectroscopy (FTIR) analysis. The efficiency of the synthetized nanoparticle in displacing heavy oil is initially assessed using an oil–wet glass micromodel at ambient conditions. Nanofluid samples with various concentrations (0.05 wt % and 0.5 wt %) dispersed in a water base fluid with varied salinities were first prepared. The prepared nanofluids provide high stability with no additive such as polymer or surfactant. Before displacement experiments were run, to achieve a better understanding of fluid–fluid and grain–fluid interactions in porous media, a series of sub-pore scale tests—including interfacial tension (IFT), contact angle, and zeta potential—were conducted. Nanofluid flooding results show that the nanofluid with the medium base fluid salinity and highest nanoparticle concertation provides the highest oil recovery. However, it is observed that increasing the nanofluid concentration from 0.05% to 0.5% provided only three percent more oil. In contrast, the lowest oil recovery resulted from low salinity water flooding. It was also observed that the measured IFT value between nanofluids and crude oil is a function of nanofluid concentration and base fluid salinities, i.e., the IFT values decrease with the increase of nanofluid concentration and base fluid salinity reduction. However, the base fluid salinity enhancement leads to wettability alteration towards more water-wetness. The main mechanisms responsible for oil recovery enhancement during nanofluid flooding is mainly attributed to wettability alteration toward water-wetness and micro-dispersion formation. However, the interfacial tension (IFT) reduction using the iron-carbon nanohybrid is also observed but the reduction is not significant.

## 1. Introduction

Secondary or tertiary recovery, are common terms used for methods to increase crude oil production from an oil field. Since the increasing demand for energy forces maximum oil extraction from reservoirs before abandonment, enhanced oil recovery is of major importance in the oil industry. During oil production, a pressure drop usually occurs in most oil reservoirs, which prevents the production of oil naturally. Therefore, various methods have been developed to improve and enhance oil production, which are classified into three major categories: primary, secondary, and tertiary. In the primary stage, oil production occurs due to the natural energy existing in a reservoir. The source of the natural energy is gas cap drive, aquifer drive, solution gas drive, rock and fluid expansion, and gravity segregation. In the secondary stage, gas or water injection increases the natural energy and thus the oil recovery. In the tertiary stage, oil production is enhanced using the injection of chemicals such as surfactants and polymers; miscible gas and thermal energy displace further oil from the reservoir after the secondary stage. To enhance oil recovery, nanotechnology has been recently considered in the oil industry. Since chemical flooding is expensive, as well as other factors such as formation damages, nanomaterials have attracted additional interest for enhanced oil production. Nanoparticles change the wettability of the reservoir rocks, increasing the viscosity of the injected fluid and decreasing the interfacial tension between oil and the injected fluid, leading to greater oil production. Due to the small size of these materials, they easily infiltrate into the pore space and therefore displace the oil existing in the pore space without being trapped. In addition, nanomaterials are typically more resistant to degradation under high pressure and temperature. Therefore, in recent years, with the advancement in nanotechnology and realization of its potential capabilities, many researchers have conducted studies on the use of nanoparticles to enhance oil production [1,2]. Particles with a size between 1 nm and 100 nm are classified as nanoparticles. Major EOR mechanisms of nanofluid flooding including wettability alteration to more water-wet, interfacial tension (IFT) reduction, the decrease of the oleic phase viscosity, disjoining pressure, and preventing asphaltene precipitation have already been investigated in the literatures. The general mechanism of wettability alteration has been well studied and reported by recent studies [1,3]. The IFT reduction between oil and injected fluid is one the main effective mechanisms relevant to enhanced oil recovery. Some types of nanoparticles such as $SiO_{2}$, polymer coated nanoparticles, ferrofluid, hydrophobic and lipophilic polysilicon nanoparticles (HLPN) have been considered as a potential agent to reduce the IFT between oleic phase and injected fluid into a core or micromodel [4,5,6,7]. The reduction of oil viscosity is another mechanism which increases the oil production during nanofluid flooding. Ogolo and coworkers [4] observed that ${\mathrm{Al}}_{2}O_{3}$ nanoparticles dispersed in brine or distilled water provides high oil recovery. They found that an ${\mathrm{Al}}_{2}O_{3}$ nanofluid is able to reduce the IFT between oil and oleic phase as well as reduce oil viscosity. However, oil viscosity reduction is the dominant mechanisms. It has also been observed that CuO nanoparticles decrease the oil viscosity as well as increasing the injected gas (${\mathrm{CO}}_{2}$) content during foam injection [8]. ${\mathrm{Fe}}_{2}O_{3}$/${\mathrm{Fe}}_{3}O_{4}$ is another type of nanoparticle which leads to an increase of oil recovery by the decrease of oil viscosity, provided it is dispersed in brine [4]. Nickel oxide (${\mathrm{Ni}}_{2}O_{3}$) nanoparticles are an effective agent for heavy oil recovery since they decrease the viscosity of the oleic phase during the flooding process [4]. To detach the oil from the surface, disjoining pressure is one of the driving forces forms at the interface between displacing and displaced phases (i.e., nanofluid and oil, respectively) and solid substrate [9]. Wasan and Nikolov [9] assumed the origin of the disjoining pressure is a wedge-shaped film at the oil–matrix interface which is formed by nanoparticles. The magnitude of the disjoining pressure is related with the wedge film thickness and nanoparticle structure [9,10]. Zhang and coworkers [11] have verified that the disjoining pressure is the main mechanism in oil displacement in their nanofluid flooding experiments. A few studies have proved that nanoparticles have the potential to prevent asphaltene precipitation [12]. Asphaltene molecules absorbs the nanoparticles existing in the nanofluid and therefore asphaltene aggregation in the pore space drastically decreases [13]. However, many of the detailed mechanisms of nanofluid flooding remain unresolved and research in this area is ongoing. Thus, to address the interaction between nanomaterials, rock surface, and oleic phase, more research is needed.

As stated above, different nanomaterials have been used in enhanced oil recovery studies. Li and Torsæter [14] carried out an experimental investigation of EOR mechanisms for nanofluid at the micromodel scale. They used both silica nano-structured particles and colloidal silica and found out that nanoparticles have the ability to reduce the IFT between oil and water as well as contact angle, to make solid surfaces more water wet. Their observations have also revealed that an increase of nanoparticle concentration increases the oil production by emulsification and reduction of IFT. Wang and coworkers [15] investigated the effect of different hydrophobic and hydrophilic silica nanoparticles, dispersed in water with the help of surfactants, on enhanced oil recovery. They found that the solid surface absorbed hydrophobic nanoparticles easier than hydrophilic nanoparticles, while hydrophilic nanoparticles were more easily adsorbed on the oil surface than hydrophobic nanoparticles. Parameters involved in the disjoining pressure mechanism, such as reduction IFT and wettability alteration, have also been studied [16]. Lipophobic and hydrophilic (LHP) silica nanoparticles with different concentrations were introduced to brine and it was observed that IFT decreases as the nanofluid concentration increases— i.e., the higher the concentrations of nanofluids, the lower the IFT [16]. However, as nanofluid concentration increases the impairment of porosity and permeability in Berea sandstone core plugs increases and therefore will not give additional oil recovery in a low-permeability reservoir. LHP silica nanoparticles will also alter the surface wettability to be more water-wet [16]. Hendraningrat and Torsæter [17] investigated the effect of a stabilizer on silica-based nanofluid stability and oil production during coreflooding process. They observed that a non-toxic stabilizer, polyvinylpyrrolidone (PVP), improves the stability of silica-based nanofluids dispersed in synthetic seawater at a particular time and temperature and therefore the oil recovery increases with the higher stability of the nanofluid. Corredor and coworkers [18], using different nanoparticles including $TiO_{2}$, $Al_{2}O_{3}$, in-situ prepared $Fe{\left(OH\right)}_{3}$ and surface-modified $SiO_{2}$ nanoparticles investigated the performance of nanopolymer (xanthan gum) flooding to enhance heavy oil recovery. Their results show that as nanoparticles concentration increases the cumulative oil recovery increases between 3% and 9%, and between 1% and 5% at 0 wt % and 0.3 wt % NaCl, respectively. Mohmmadalinejad and coworkers [19] investigated the formation damage during displacement of crude oil by silica nanofluids using two identical glass micromodels with water-wet and oil-wet surfaces in the presence and absence of connate water saturation. Their observations indicate that oil production decreases drastically after the formation damage.

To the best of the authors’ knowledge, there are no published investigations on the effect of iron-carbon nanohybrid particles in the presence of brine with varied salinities as a secondary recovery method. The investigated iron-carbon nanohybrid is a kind of smart nanoparticle since it is sensitive to the ultra-violet (UV) light and hence it can be used as a tracer. Due to its magnetization property, the investigated nanohybrid can be separated by a magnetic separator in the well outlet and hence there will be no environmental effect. Therefore, in the current study, the performance of varying iron-carbon nanohybrid concentrations in the presence of various amounts of salt on the wettability alteration, IFT reduction, forming micro-emulsion, and also improving the oil recovery factor has been investigated. For this purpose, a clean micromodel with the heterogenous pattern is used as the porous media to observe the main effective mechanisms on heavy oil displacement in the porous media. To understand the role of fluid–fluid and solid–fluid interactions, zeta potential, interfacial tension (IFT), and contact angle experiments were also carried out. The results obtained at the laboratory scale may be useful to predict the capability of the investigated nanohybrid for field applications.

## 2. Materials and Methods

### 2.1. Materials

#### 2.1.1. Chemicals

Ferrous chloride (FeCl_2_ 4H_2_O), Ferric chloride (FeCl_3_ 6H_2_O), aqueous ammonia solution (NH4OH 30%wt), and citric acid were all purchased from Merck. All materials were used as received without further purification and deionized water was used in all steps of the experimental tests.

#### 2.1.2. Synthesis of Carbon Nanoparticle

Carbon nanoparticles were synthesized via the hydrothermal method with citric acid ($C_{6}H_{8}O_{7}$) as a carbon source. Briefly, 2 g of citric acid mixed with 20 mL of deionized water and the obtained mixture was stirred vigorously for 15 min and then transferred into a Teflon autoclave, heated to 200 °C for 8 h, and cooled to room temperature.

#### 2.1.3. Synthesis of Fe_3_O_4_/Carbon Nanohybrid

99.5 mg of ${\mathrm{FeCl}}_{2}.4H_{2}O$ with 270 mg of ${\mathrm{FeCl}}_{3}.6H_{2}O$ was dispersed in 30 mL deionized water and the mixture was stirred for 5 min. Then 30 mL carbon nanoparticle (1 mg/mL) was added to the mixture at room temperature. During severe agitation, 2 mL ammonia solution was added gently for making the color change from light brown to dark brown and finally to black. The mixture was heated to temperature of 80 °C and was kept in this temperature for 1 h by severe agitation. After cooling the mixture, the product was washed by deionized water several times until the pH of 7 was reached and then the solid material was separated from water using a magnet.

#### 2.1.4. Nanoparticle Characterization

**Figure 1:**
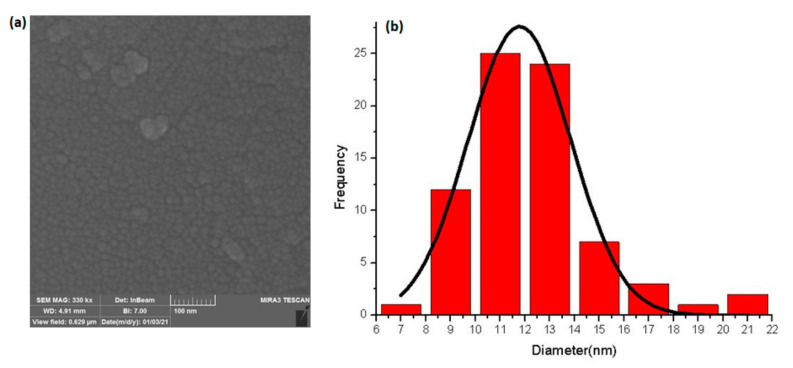
(**a**) FESEM images of iron-carbon nanohybrid (**b**) the particle size distribution of nanohybrid particles.

Field-emission scanning electron microscope (FESEM) is a microscope that works with electrons (particles with a negative charge) instead of light. A FESEM image is used to visualize very small topographic details on the surface or entire or fractioned objects. Figure 1a shows the FESEM image of the investigated nanoparticle. As can be clearly seen in Figure 1a, the size of nanoparticles is rather uniform but most of the nanoparticles almost possess spherical shape. In addition, analysis of the particle size distribution revealed that the average particle size of the used nanohybrid is 11.78 nm. It shows that nanoparticles are distributed most likely around 25% in range 10–12 nm.

**Figure 2:**
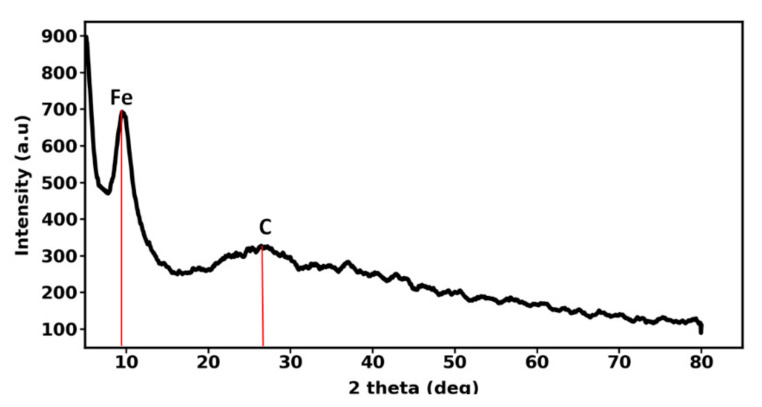
XRD pattern of iron-carbon nanohybrid.

Powder XRD patterns were collected by a Philips PW1730 X-ray diffractometer in the angle range of 5–80°. Figure 2 shows the XRD pattern of iron-carbon nanohybrid. The broad diffraction peak which has been located at 2θ ~ 26° suggests that the iron-carbon nanohybrid has an amorphous nature. Moreover, this peak further indicates the existence of carbon atom in the iron-carbon nanohybrid. The peak located at 2θ ~ 10° is representative of the iron atom.

**Figure 3:**
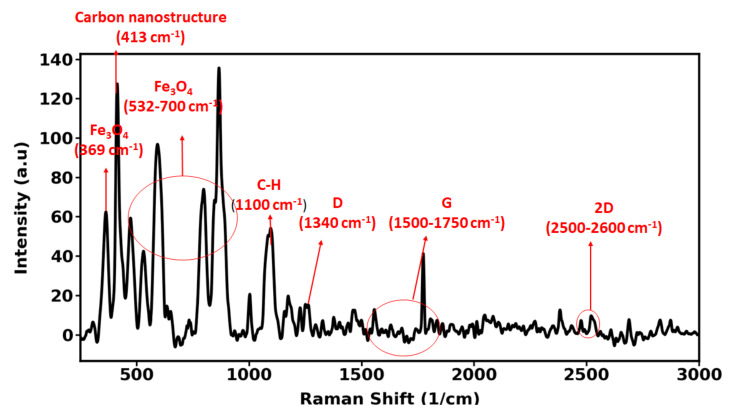
Raman spectroscopy of iron-carbon nanohybrid.

Raman spectroscopy is used as a powerful method for characterization of carbonic nanostructure. The Raman spectrum of the iron-carbon nanohybrid is shown in Figure 3. Accordingly, the appearance of three distinctive peaks at around 1340, 1773, and 2600 cm^−1^ can be attributed to D, G, and 2D bands, respectively. The G band is commonly appeared in all Sp^2^ carbon allotropes. While the intensity of the D band shows that defect structures have been partially created in synthesis procedure which is reported very low for the investigated nanohybrid. A peak at around 413 cm^−1^ confirming the existence of carbon nanostructure. Magnetite is another mineral which exists in the nanohybrid studied here. Since magnetite is prone to transformation if laser power is too high there are varied bands for magnetite mineral. de Faria et al. (1997) state three bands for magnetite: at 300, 532, and 661 cm^−1^.

#### 2.1.5. Oil Specifications

A polar heavy crude oil was obtained from one of the Iranian oil reservoirs and used in this study as an oleic phase. This oil is characterized by large amount of asphaltene (9.7%) and high and low base (TBN) and acid numbers (TAN), respectively. TBN and TAN were measured based on ASTM 148 D2896 and ASTM D664 standard procedures, respectively. The heavy crude oil physical properties and SARA analysis are listed in Table 1. It should be noted that the dead heavy crude oil was used for all the experiments in this study.

#### 2.1.6. Brine Solutions

Three types of aqueous solutions including synthetic seawater (SW), 2 times diluted version of SW (2SW), and 100 times diluted version of SW (100SW) were used in this study for dynamic oil displacement, IFT, and contact angle tests. Furthermore, samples 2SW and 100SW were prepared using dilution by adding deionized water. The brine solutions properties are shown in Table 2.

### 2.2. Methods

#### 2.2.1. Colloidal Stability

The stability of nanofluids has been reported by the Derjaguin–Landau–Verwey–Overbeek (DLVO) and the non-DLVO theories [20]. The DLVO theory expresses that the colloidal stability is due to the balance between the attractive forces (van der Waals forces) and the electrostatic repulsive forces. The repulsive forces prevent nanoparticles from approaching each other, thus resulting in a stable nano-solution. However, the attractive forces lead to the nanoparticles verging together, which results in the aggregation of particles. The source of the attractive forces is the electrical and the magnetic polarization properties of the ions of nanoparticles. The stability of nanofluids depends on the surface charge of nanoparticles and base fluid salinity—i.e., the higher surface charges of nanoparticles provide the more stable solution while the higher salinity reduces the stability of the nanofluid [21]. As well as the DLVO forces, the non-DLVO forces—including the stearic, magnetic forces, and hydration forces—are also effective on the stability of the solution [20]. Stearic forces are due to the existence of organic materials (such as surfactants) in the solution. Particles such as iron, nickel, cobalt, and manganese are able to form a magnetic dipole moment in the absence of applied magnetic field which is the origin of the magnetic forces. Hydration forces are due to the hydrogen bonding that is formed with water molecules, as well as with other molecules. Hence, to approximate hydrophilic particles close together, it is required that the hydrogen-bonding network between them has been broken. Breaking the hydrogen-bonding network between particles leads to increasing the enthalpy, consequently the hydration force is generally a repulsive force.

A fundamental and effective mechanism of the electrostatic stabilization of colloids is the formation of the electrical double layer at the interface between particle surfaces and bulk fluid. The charged particles surfaces dispersed in an ionic solution attract the opposite charges present in the solution and form a thin layer which is firmly bound to the particle surface, named the Stern layer. The second layer, named the diffuse layer, is loosely associated with the particle surfaces which is made of free ions that move in the fluid. The Stern and diffuse layers create an electrical double layer. The zeta potential is defined as a potential difference between the bulk fluid and the layer of fluid containing the oppositely charged ions that is bound to the particle surface. To evaluate and predict the stability of the solution and the formation of aggregates, zeta potential measurements are generally used. However, the formation of aggregates is not the function of zeta potential, only because of the presence of other interaction between particles and the base fluid such as van der Waals forces, hydrophobic, hydrophilic, structural, and steric interactions [22]. Zeta potential is a property used to detect the surface charge of colloidal solutions. Therefore, pH values, ionic strength of bulk liquid, and temperature are effective parameters of the zeta potential value.

#### 2.2.2. Fourier Transform Infrared Spectroscopy (FTIR)

Fourier transform infrared spectroscopy (FTIR) is a method that is used to obtain infrared spectrum of absorption, emission, and photoconductivity of solid, liquid, and gas to detect different functional groups of the functionalized nanoparticles, organic, inorganic, and polymeric materials using infrared light for scanning the samples. Changes in the material compound are detectable with the alterations in the spectrum pattern of absorption bands. Therefore, FTIR technology is useful for identification and characterization of unknown materials, detecting contaminants in a material, finding additives, and identifying decomposition and oxidation.

#### 2.2.3. Viscosity of the Nanofluid

The mobility ratio, as a function of the effective viscosity and relative permeability of the injected fluid and oil ($k_{i}\mu_{o}/k_{o}\mu_{i}$), is an important parameter for characterizing the EOR process. Injecting a fluid such as water, ${\mathrm{CO}}_{2}$ or chemical as the EOR agent leads to a higher mobility ratio since the viscosity of the injected fluid is lower than that of the oil. A high mobility ratio between the oil and the displacing fluid causes the injected fluid to finger through the reservoir and leave a significant amount of oil in the reservoir, resulting in low recovery. A higher sweep efficiency is produced as a result of a low mobility ratio. The mobility ratio has a positive and negative relationship with the oil and injected fluid viscosities, respectively. Hence, oil viscosity reduction or an increase of the injected fluid viscosity result in a lower mobility ratio and thus higher oil production. The nanofluid is an effective EOR agent since nanofluid viscosity is much higher than the viscosity of the traditional injected fluid such as water. In this study, the viscosity of the nanofluid was measured at 25 °C using a MCR501 rheometer (Anton Paar GmbH, Ostfildern, Germany). The shear rate was varied from 0.01 to 100 $s^{-1}$.

#### 2.2.4. Interfacial Tension (IFT) Measurement

**Figure 4:**
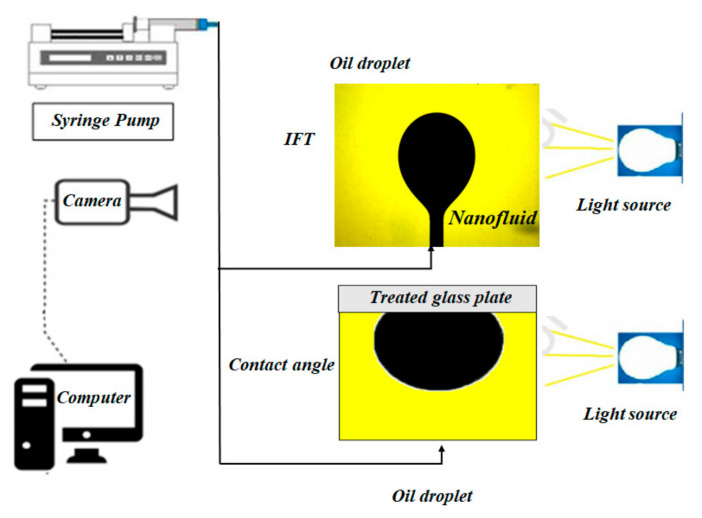
Schematic illustration of the used setups for IFT and contact angle measurements.

Interfacial tension (IFT) is considered as the indication of the energy at the interface of two immiscible fluid and so is a fundamental parameter for the EOR process. The successful agents in EOR processes—such as nanofluids, surfactants, polymer, and low salinity water—tend to reduce the IFT between oil and the displacing fluid and thus produce more residual oil. IFT measurement can be performed optically using the pendant drop shape analysis. In this method, the rising pendant drop (crude oil) is introduced in an immiscible fluid (aqueous phase). In this study, the IFT setup consists of a 50 $cm^{3}$ transparent chamber, a syringe pump, a digital camera with macro lens and a computer to monitor and save the images. The captured images were analyzed using an image processing code based on Laplace equation compiled in LabVIEW. Figure 4 illustrates the schematic diagram of the prepared setup for IFT and contact angle measurement experiments. Each IFT test was repeated four times to assess the stability of IFT values and the mean IFT value was reported.

#### 2.2.5. Contact Angle Measurement

The interaction between fluids and the rock surface has a significant effect on the capillary pressure, relative permeability, fluid displacement flooding behavior, and electrical properties [23]. Therefore, to investigate the interactions between the rock surface and pore fluids in both sandstones and carbonates, a large number of studies have been carried out by measuring the fluid–solid contact angle [24,25,26,27,28,29,30,31]. In this study, wettability alteration in oil-wet surfaces has been investigated through contact angle measurements because surface wettability changes from oil-wet to water-wet has a significant influence on residual oil saturation. To investigate the effect of the nanofluid on wettability changes, nine glass plates were prepared. In order to alter the wettability of the glass plate from the naturally water-wet to oil-wet conditions, the plates were immersed in a solution of 2 wt % trichloromethyl silane (TCMS) in dehydrated toluene and methanol for 15 min, respectively. Following this treatment, the prepared glass plates become hydrophobic. After that, six nanofluids including two different nanoparticle concentrations (0.05 wt % and 0.5 wt %) in three types of aqueous solutions including seawater (SW), twice diluted SW (2SW), and 100 times diluted SW (100SW) were prepared. Then, six oil-wet glass plates were soaked in the prepared nanofluids for 10 days. Three oil-wet glass plates were also soaked (for 10 days) in the base fluid—i.e., SW, 2SW, and 100SW. To assess the effect of different nanofluids as well as different brines on the surface wettability alteration, the Sessile drop method was used. The contact angle measurements were carried out using a simple and accurate setup in the ambient condition. When the aging time (10 days) was passed, the glass plates were placed on the top of transparent cell filled with related nanofluids. The oil droplet was injected to the bottom of the cell using a syringe pump. Since the density of the crude oil and brine is different, the oil droplet moves up and adheres to the glass surface. Figure 4 illustrates the schematic diagram of the prepared setup for contact angle experiments.

#### 2.2.6. Displacement Experiments

**Figure 5:**
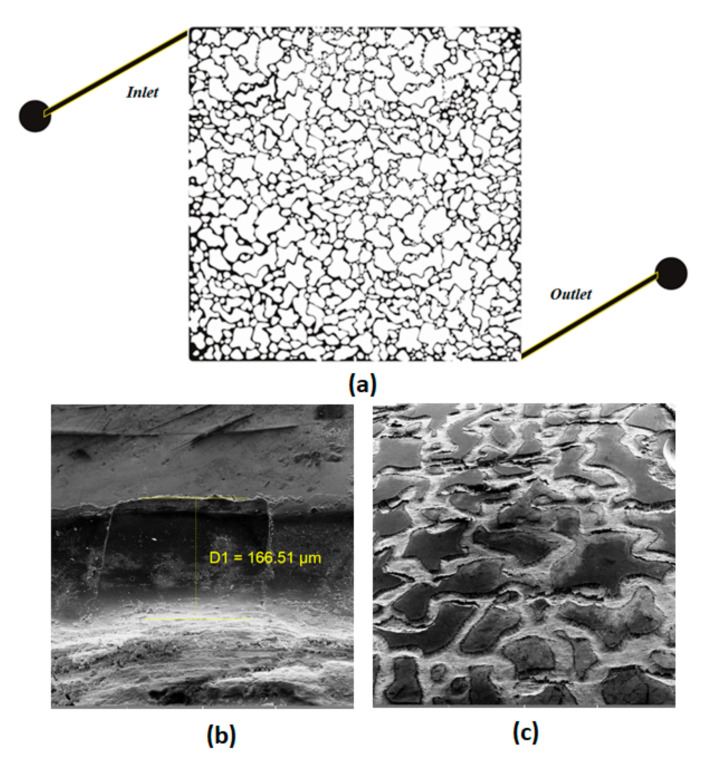
(**a**) The heterogenous network pattern of the investigated micromodel. The SEM images of etched channels: (**b**) top view, (**c**) tilted view.

A glass micromodel with heterogeneous pore structures was used as a porous media to investigate the volumetric sweep efficiency during the nanofluid injection visually. The pattern was designed by means of a graphical software and then it was engraved onto a glass plate using laser etching technology. The network pattern is illustrated in Figure 5a.

The porosity of the porous media was measured by image analysis after injecting oil to the micromodel to differentiate between grain (the white color) and pore (filled by oil with the black color) within the micromodel. The absolute permeability is one of the fundamental petrophysical properties of the porous media which provides the capacity of the medium to transmit fluids. It can be viewed as a function of the pressure difference across the porous medium at different flow rates. The permeability of the investigated micromodel was measured by injecting a fluid, with known viscosity, through the micromodel at different flow rates and monitoring the pressure drop across the micromodel using

(1) $$k=\mu \frac{qL}{A\Delta P}$$

where *k* is the absolute permeability ($m^{2}$), μ is the viscosity of the injecting fluid (Pa. s), *q* is the flow rate ($m^{3}/s$), *L* is the length of the porous medium from inlet to outlet (m), *A* is the cross-sectional area perpendicular to the flow ($m^{2}$), and is ΔP the pressure difference across the porous media (Pa).

The pore volume of the micromodel can be determined if the average etched depth and the cross-section area perpendicular to the flow are known. The SEM images of the investigated pore network, shown in Figure 5b,c, provide the average etched depth.

The pore size distribution of the porous medium used in this study is illustrated that the average pore radius is 65 µm. The physical and petrophysical properties of the micromodel are presented in Table 3.

**Figure 6:**
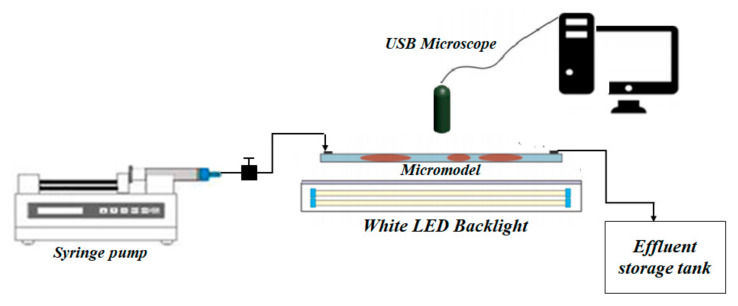
Schematic representation of fluid injection systems including a glass micromodel, a syringe pump for nanofluid and oil injection, USB digital microscope for continuous recording of images, a computer for data acquisition and a white LED backlight for diffusion light source to illuminate the micromodel.

The experimental setup comprises of a syringe pump system capable of injecting fluids into the micromodel at very low rate (ranging from 0.0001 to 3.0 $cc.min^{-1}$). To clean the micromodel, toluene was injected to the micromodel followed by distilled water by means of a syringe pump. The syringe pump was connected to the micromodel. A backlight source was placed under the micromodel, while a high-resolution video camera was installed above the micromodel and connected to a computer to schedule imaging times to record the variations of fluid saturations within the micromodel. To collect effluent, a storage tank was connected to the outlet port of the micromodel. Figure 6 shows the schematic elements of the apparatus.

To evaluate the capability of the iron-carbon nanohybrid as an EOR agent, six different displacement experiments were designed and conducted on an oil-wet micromodel. All displacement experiments were performed under the same conditions of porosity and permeability. All nanofluid solutions were freshly prepared just before being used to avoid any effect of air exposure, which may alter the surface tension. To alter the glass surface wettability from naturally water-wet to oil-wet, special treatment by chemical agents was carried out as follows [32,33]. (1) Injecting sodium hydroxide into the pore network for an hour followed by washing the micromodel with distilled water; (2) placing the micromodel in an oven at 200 °C for 20 min; (3) injecting a dilute solution of 2 wt % trichloromethyl silane (TCMS) and dehydrated toluene into the micromodel for 15 min followed by the washing micromodel with methanol; (4) placing the micromodel in an oven at 100 °C for an hour to stabilize the siliconizing fluid.

After providing the oil-wetting process of the micromodel, it was fully saturated with multiple pore volumes of the crude oil with the composition reported in Table 1 and then the flooding proceeded with the flow rate based on capillary number as

(2) $$Ca=\frac{\mu \nu }{\sigma }$$

where Ca is the capillary number, μ is the viscosity of the displacing fluid, ν is the velocity of the displacing fluid, and σ is the IFT between displacing and displaced fluid, i.e., nanofluid and oil, respectively. To simulate the flow pattern and transport through porous media and achieve laminar flow conditions through the micromodel, it is required that the capillary number remains below the threshold of $10^{-5}$. Based on the measured IFT values between different displacing and displaced fluids (nanofluids and crude oil, respectively) and the viscosity of nanofluids, the flow rate was calculated to be 1 $µL\min^{-1}$ which provides the capillary number below the threshold of $10^{-5}$. All the experiments were performed at ambient temperature and pressure. A description of each experiment is presented in Table 4.

## 3. Results and Discussion

### 3.1. Colloidal Stability

**Figure 7:**
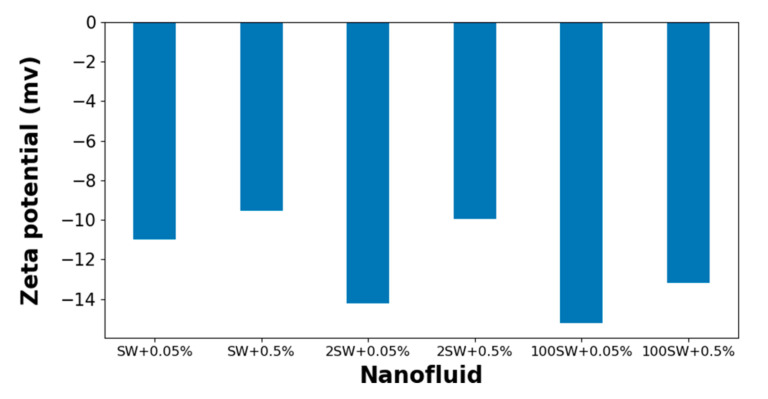
Changes of zeta potential between nanofluids with different salinities and concentrations.

In order to determine the surface charge of nanoparticles in colloidal solution, zeta potential is an analytical measurement that is generally used. The nanofluid samples (listed in Table 4) were used to measure the zeta potential. As is shown in Figure 7, all samples showed negative zeta potential values. As explained earlier, zeta potential is affected by the properties of the nanoparticles, as well as the nature of the bulk fluid, such as pH and ionic strength. It was observed that the presence of salt in the dispersant medium decreases the absolute value of zeta potential of the nanofluids, which is consistent with the former works [21], since that higher salinity of the bulk fluid caused the shrinkage of the double layer. The nanofluid used in this study seems stable despite the low zeta potential value. The same observation was reported by other studies [18,34,35,36]. It should be noted that zeta potential value is not the only indication of nanoparticle stability since addition to the DLVO forces there are hydration forces between nanoparticle surfaces preventing the particle coagulation.

### 3.2. FTIR Measurements of the Carbon Nanohybrid

**Figure 8:**
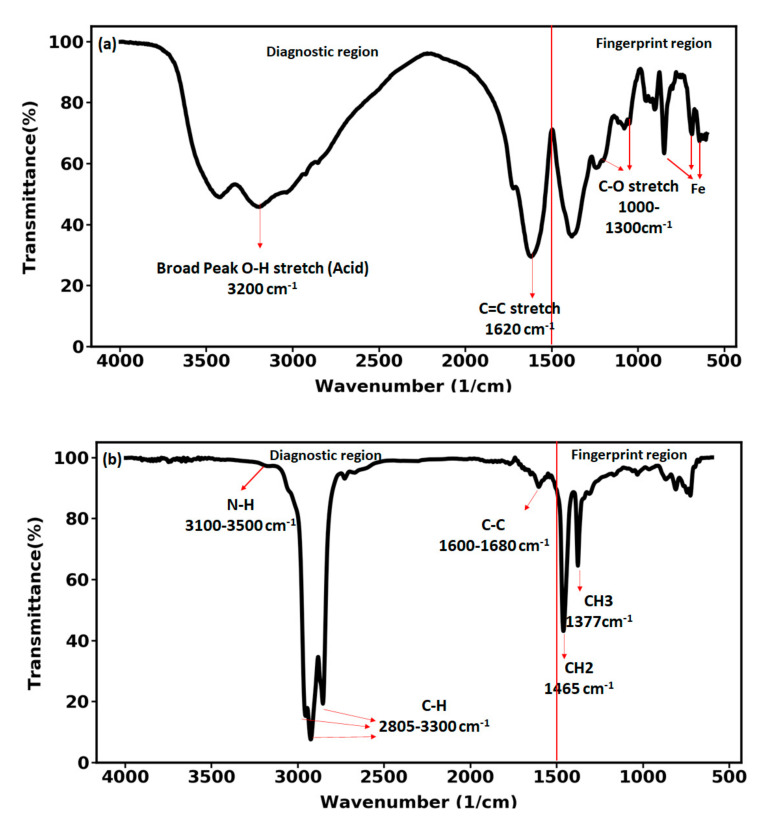
FTIR spectrum of (**a**) the iron-carbon nanohybrid (**b**) the crude oil.

FTIR spectroscopy is used mostly to identify the functional group of the molecules. The FTIR spectrum includes two regions: the diagnostic region which lies above the wavenumber of 1500 cm^−1^ and the fingerprint region below the wavenumber of 1500 cm^−1^. In the fingerprint region, the spectrum is unique for every compound. This region can also be used to identify a compound or check if a compound is pure. Therefore, to identify the functional groups of the functionalized nanoparticles the diagnostic region of the FTIR spectrum was investigated (Figure 8). As can be seen in Figure 8a, there is a wide stretching hydroxyl band (O-H) around the wavenumber of 3200 cm^−1^. Since this peak is broad with high absorptions and also joins up with the C-H stretching in the range of 2850–3300 cm^−1^ it can be attributed to the carboxylic acid available in the nanoparticle. The peak at 1620 cm^−1^ corresponds to the C=C stretching vibrations. Similar to the C=O stretching vibration, C=C stretching is sharp and thin but it is much higher up than that. The absorption peaks between wavenumber of 1000–1300 cm^−1^ indicate the C-O stretching vibrations. Finally, nanohybrid provided two peaks at 644 and 688 cm^−1^ with a rather strong peak at 848 cm^−1^ which are the indication of existence of iron in the investigated nanohybrid. The FTIR spectrum of the crude oil is shown in Figure 8b. The wavelength 1600.8 cm^−1^ is the representative of alkane group (C-C). Additionally, at wavelengths ranging from 2850 cm^−1^ to 2950 cm^−1^ including C-H stretching vibrations. The absorption peak at the wavenumber 3184 cm^−1^ is related to N-H (cyclic aromatic nitrogen compound is in the ranges of 3100–3500 cm^−1^). In the spectral region of 1375–1465 cm^−1^, two strong peaks are observed, which are attributed to $CH_{2}$ and $CH_{3}$ bending group. It has been experimentally reported that these polar bends play an important role in altering wettability [37,38].

### 3.3. Viscosity Measurements

**Figure 9:**
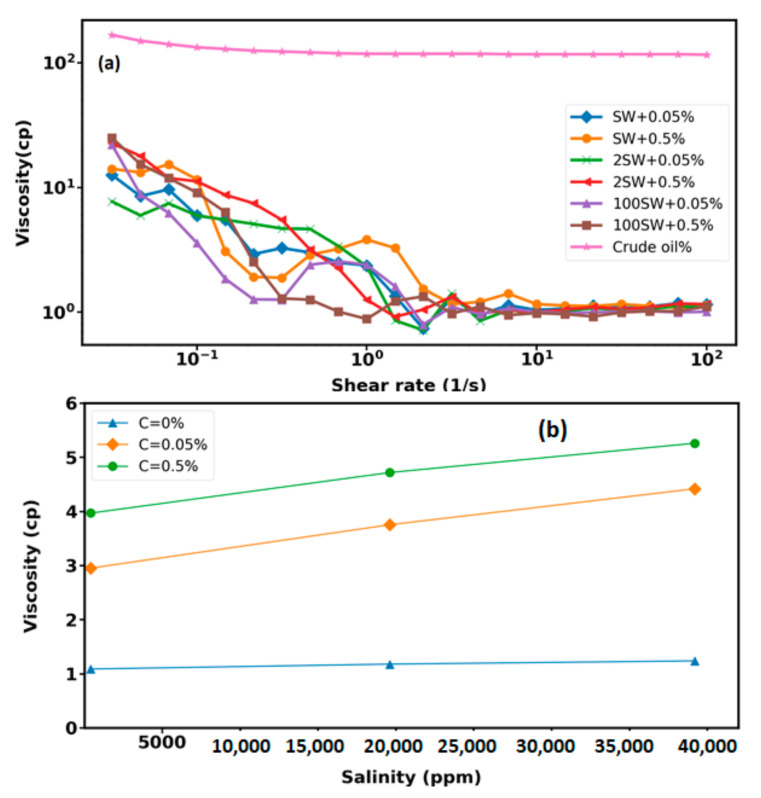
(**a**) Dynamic viscosity of the iron-carbon nanohybrid and the crude oil as a function of shear rate. (**b**) Average viscosity variations with nanoparticle concentration and base fluid salinity at ambient condition.

To enhance oil production, the optimum concentrations of the displacing fluids in EOR applications is required. Therefore, the rheological measurements are fundamental, since the viscosity of the displacing fluid affects the mobility ratio and then has an important role in the enhanced oil recovery process. Figure 9a shows the rheological behavior of the investigated crude oil and nanoparticles with different nanoparticle concentrations and base fluid salinities at 25 °C between 0.01 and 100 $s^{-1}$. The effective factors on the dynamic viscosity of the investigated nanofluids are the nanoparticle concentration, base fluid salinity, and the shear rate. As is observed, the viscosity of the nanofluids and the crude oil decreases with increasing the shear rate, until it reaches a point where the magnitude of viscosity value is independent of the shear rate. The effect of nanoparticle concentrations and the salinity of the base fluid on the effective dynamic viscosity is shown in Figure 9b: The average viscosity increases with the enhancement of base fluid salinity and nanoparticle concentration. Abbas and coworkers [39] showed that the presence of salt ions can increase the viscosity of nanofluids.

### 3.4. Interfacial Tension (IFT) Measurement

**Figure 10:**
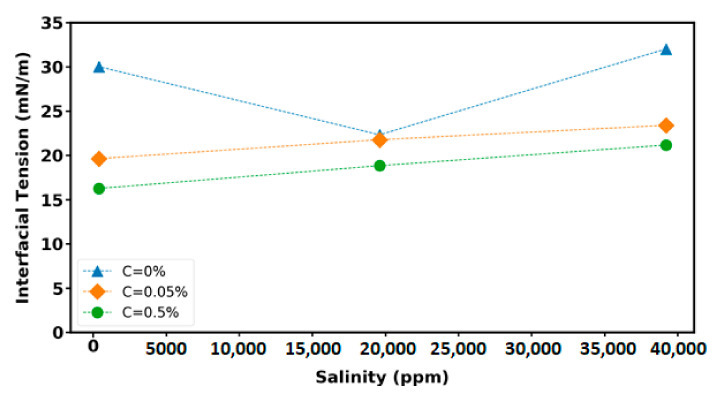
Interfacial tension (IFT) variations as a function of base fluid salinity and nanoparticle concentration.

In order to determine the fluid distribution and transportation in porous media, the definition of the IFT value betw een displacing and displaced fluid (i.e., the nanofluid and the crude oil, respectively) is required. IFT reduction is one of the fundamental EOR mechanisms. In this study, the IFT measurements between the investigated nanofluid samples and the crude oil was carried out. Figure 10 illustrates the IFT variations between nanofluid, with different nanofluid concentrations and varied base fluid salinity, and the heavy crude oil. As is observed, the IFT values increased with the enhancement of the base fluid salinity and the reduction of the nanofluid concentrations. Therefore, the lowest IFT value is related to the sample with the highest nanofluid concentration (C = 0.5%) and the lowest salinity (100SW); however, the effect of nanofluid salinity on IFT reduction is not significant. The absorption of nanoparticles at the interface between nanofluid and the crude oil can change the IFT value of the nanofluid-oil systems. As the nanofluid concentration increases, the repulsive electrostatic forces between nanoparticles causes the nanoparticles diffuse from the aqueous brine solution to the nanofluid-oil interface. Consequently, the interface is more saturated with nanoparticles and IFT between the injected fluid and the crude oil decreases. Therefore, the nanofluid concentration is an effective parameter on the IFT value and it can be inferred that the nanofluid has the potential to produce more oil from the reservoir in comparison to the brine. It is also noted that highly active and stable nanoparticles reduce the interfacial energy at the interface between nanofluids and crude oil, which decreases the interfacial tension.

However, the IFT measurements between crude oil and base fluid (C = 0%) have demonstrated that the lowest IFT value corresponds to the twice diluted version of SW (i.e., 2SW). Other studies [40,41,42,43] have reported the same trend as Figure 10 (for C = 0%) in which the IFT value decreases with the reduction of the brine salinity until reaching a specific salinity (for this study 2SW) and then increasing the IFT value with the decrease of salinity further than the specific salinity. The crude oil contains asphaltenes, resins, and waxes which are considered as the surface-active agent of crude oil and act like a natural surfactant. Previous research found that the natural surfactants in the crude oil (i.e., asphaltenes, resins) have a significant effect on the variation of IFT value of crude oil–brine system [40,42,44]. The distribution of asphaltene and resin in the oil phase alters with the enhancement of salinity since the diffusion of asphaltene and resin from the aqueous brine solution to the interface occurs due to the ions of brine. Therefore, the IFT value of the crude oil–brine system decreases due to the presence of the natural surfactant present in the crude oil at the interface between crude oil and brine. However, the solubility of the crude oil components in aqueous phase decreases with the increase of salinity and so IFT value increases since the crude oil surface active particles move back to the oil phase where they belong and eventually increase IFT. At low salinity (below 1000 ppm) natural surfactant will be diffused from the bulk to the oil–water interface with higher acceleration rate but reducing the activity coefficient of the salt prevents the salt molecules to penetrate into the crude oil bulk, causing lesser interactions of cations with asphaltene and resin at the interface, therefore, natural surfactants and cations migrate to their bulk phase where they origin and therefore the IFT increases at low salinity brine–crude oil system. Rostami and coworkers [40] using the microscopic images of the oil–brine interface with different salinities have illustrated that the amount of asphaltene particles at the interface between oil and deionized water is very low which is consistent with the highest value of the IFT while there is a great amount of asphaltene at the interface of oil and 2SW sample which is corresponded with the lowest value of the IFT.

### 3.5. Contact Angle Measurement

To quantify the effect of investigated nanoparticles with different base fluids on wettability alteration, the contact angle between oil–wet glass substrate, which was initially aged in different fluids (as listed in Table 4), and oil drop was measured. The interactions between three phases—including the nanoparticle, oil drop, and the solid surface—is related to surface wettability. The collected data show that the significant effect of nanofluid on wettability alteration from the oil-wet surfaces to water-wet surfaces.

Recent studies have shown that nanofluid is an effective EOR agent by reducing the IFT and changing the wettability alteration [45,46,47,48]. Wasan and Nikolov [9] presented the initial conceptual mechanism for wettability alteration due to nanofluid injection. According to their findings, nanoparticles form a wedge-like structure which enhances the ability of nanoparticles to detach the oil phase from the oil-wet formations. Hence, the increase of the nanofluid concentration increases the structural disjoining pressure due to the wedge-like structure, leading to wettability alteration toward water-wetness. However, the type of oil and the surface charge of nanoparticles are effective factors on the disjoining force to displace the oil from the surface. Therefore, one may conclude that the higher nanoparticle adsorption on the surface provides the higher structural disjoining force, leading to easier oil detachment from the surface.

**Figure 11:**
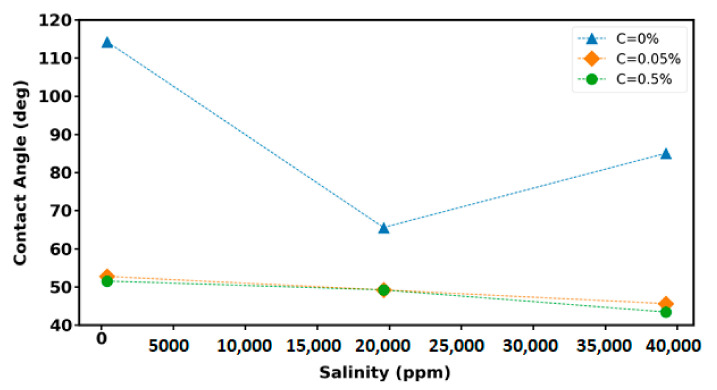
Contact angle variations as a function of base fluid salinity and nanoparticle concentration.

The contact angle measurements between crude oil and the oil–wet glass substrates aged in the base fluid (i.e., SW, 2SW, and 100SW where C = 0%) have shown that the lowest contact value corresponds to the 2SW in which the contact angle value decreases with the reduction of the brine salinity until reaching a specific salinity (for this study 2SW) and then increasing the contact angle value with the decrease of salinity further than the specific salinity (Figure 11). The same trend is reported the for the measured IFT between crude oil and base fluids (Figure 10 for C = 0%). It can be concluded that, according to Young’s equation [49], the contact angle is related to oil–water interfacial tension. Therefore, the reduction in the measured IFT between crude oil and water results in the lower contact angle between the crude oil droplet, water, the surface substrate.

### 3.6. Quantitative and Qualitative Analyses of Micro-Dispersion

Recent research [37,50] has demonstrated that when two immiscible phases, such as water and crude oil are brought in contact, micro-dispersions or microemulsions within a porous medium are formed. However, more recent studies have also shown that nanoparticles are capable of forming micro-emulsion; since particles are more strongly attached to interfaces, thus they are stable to coalescence [51,52]. There are three types of micro-dispersions including direct (oil dispersed in water, where the amount of oil is low), reversed (water dispersed in oil, where there is a low amount of water) and bi-continuous (where the amount of oil and water are equal). As mentioned earlier, crude oil contains asphaltenes, resins, and waxes which are considered as the surface-active agent of crude oil and act like a natural surfactant. The natural surfactant has two functional groups including a hydrophilic (water-soluble) or polar head and a hydrophobic (oil-soluble) or non-polar tail. The surfactant molecules (the polar component of crude oil) form a monolayer at the water–oil interface where the non-polar tails of the surfactant dissolved in the oil phase and the polar head tends to the aqueous phase. This structure is called direct micelle. Inverse micelles may form with polar heads pointing into a water core and hydrophobic tails pointing into the oil phase due to migration of the natural surfactant from the water–oil interface as result of water salinity reduction. Hence, water micro-dispersion is formed and micro droplets of the injected fluid are dispersed in oil phase and join together and form a continuous layer which is able to displace the oil present in the porous media and enhance the oil production.

**Figure 12:**
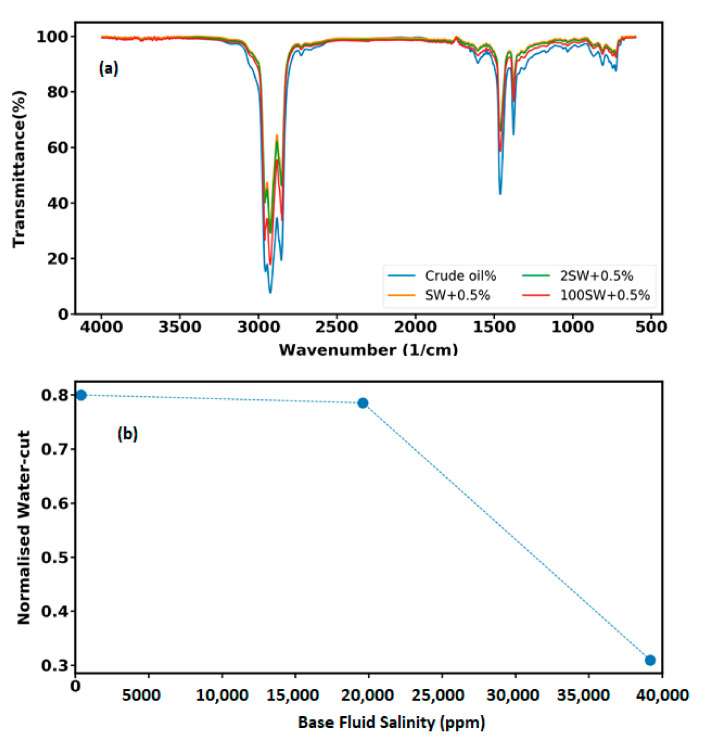
(**a**) Results of FTIR spectroscopy versus wavenumber for brine solutions with different ionic strength. (**b**) The ratio between water-cut of original crude oil and amount of water after contacting with water versus salinity level of base fluid.

The capabilities of crude oil to form micro-dispersion can be assessed using both quantitative (detection of water-cut after contacting with nanofluids) and qualitative (FTIR test) analyses. Prior to performing FTIR test, the crude oil was contacted with nanofluid with varied base fluid salinity (SW+0.5%, 2SW+0.5%, and 100SW+0.5%) for a duration of 24 h. The resultant sample transmittance is plotted as a function of wave number (600–4000 cm^−1^) for nanofluids with different salinities (Figure 12a). Lower transmittance means higher abundance of a bond; any changes in compositions of crude oil will cause variations of attenuation. By comparison of attenuation variation for the original crude oil and that after contacting with different nanofluids, it can be observed that compositional changes occur, which are indicative of micro-dispersion formation. In fact, when the crude oil is in contact with the nanofluid, active surface components of the crude oil (in the form of natural surfactant) migrate to the surface and interact with divalent cations, leading to the attenuation alteration. Therefore, it can be concluded that the investigated crude oil has the potential to form micro-dispersions even at ambient temperature.

To further investigation of liquid–liquid interaction, the amount of the spontaneous water-in-oil micro-dispersion was measured by means of distillation method (ASTM D95). The water-cut for the crude oil before contacting with nanofluids was found to be 0.025% (volume). This value was used to normalize the amount of oil water-cut after contacting with nanofluid with different base fluid salinity. Figure 12b illustrates the ratio of water-cut in oil after contacting with nanofluid solutions to that in original crude oil versus the base fluid salinity. It can be seen that as the ionic strength of the nanofluid decreases, the amount of micro-dispersion increases, which illustrates how liquid–liquid interactions depend upon the salinity level. However, for the lowest base fluid salinity (i.e., 100SW + 0.5%) the measured water-cut in comparison to the sample 2SW + 0.5% is insignificant which may be due to the significant reduction of cations available in nanofluid.

**Figure 13:**
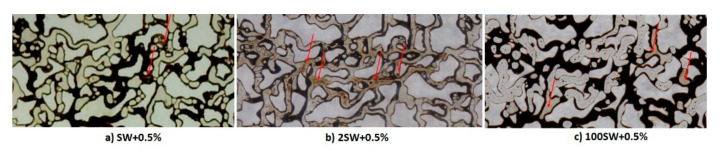
Selected sections of the oil-wet porous media during: (**a**) SW + 0.5%, (**b**) 2SW + 0 5%, (**c**) 100SW + 0.5%, flooding. The red arrows highlight the formation of micro-dispersion.

As can be seen in Figure 13, according to the results presented in Section 3.3, Section 3.4 and Section 3.5, the main mechanisms responsible for the heavy crude oil recovery enhancement during nanofluid flooding within hydrophobic porous media are proposed to be the reduction of IFT, wettability alteration, and formation of micro-dispersion.

### 3.7. Displacement Tests

**Figure 14:**
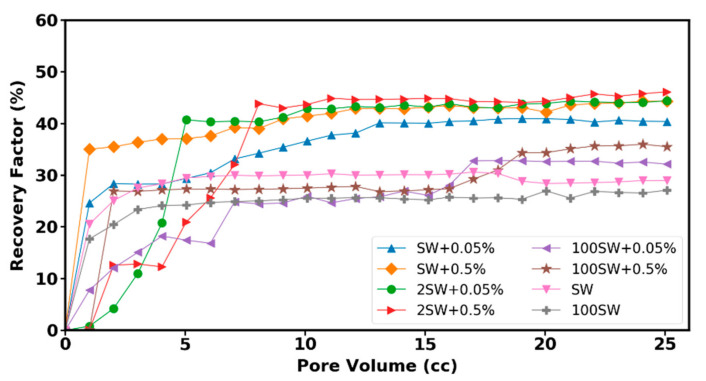
Oil recovery factor as a function of injection time for six displacing fluid for oil-wet porous media.

The effectiveness of nanofluid on enhanced oil recovery was tested with six sets of flooding experiments performed in the micromodel systems. Figure 14 shows the performance of six brine-based nanofluids (refer to Table 4) in comparison to water flooding (SW and 100SW) during injection into the glass micromodel. As can be seen, adding 0.5 wt % nanoparticle to the 2SW provides the highest oil recovery.

**Figure 15:**
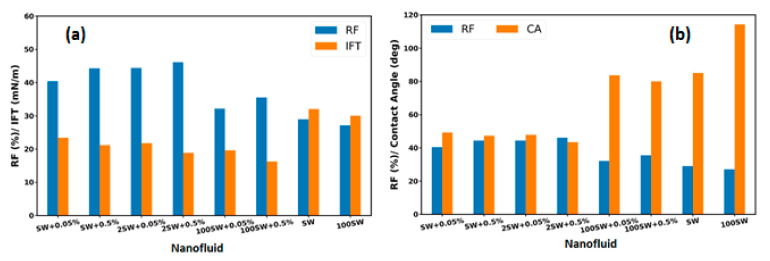
Final oil recovery: (**a**) IFT values and (**b**) contact angles for different base fluid salinities and nanofluid concentrations.

The mechanism of EOR using water-based nanofluids for the used crude oil is mainly attributed to the wettability alteration and interfacial tension reduction (as explained in Section 3.4 and Section 3.5) and micro-dispersion formation. The final oil recovery of the mentioned displacement experiments and the IFT values of the related solutions and the measured contact angle between oil droplet and treated substrate are presented in Figure 15a,b, respectively. Enhancement of the nanoparticles to the brine with different salinities improved the amount of oil recovery from 29% (test SW injection) to a value of 44% for (test SW + 0.5% injection). As is clearly observed, the solution of 2SW + 0.5% with the lowest contact angle value provides the highest oil recovery. Conversely, the highest contact angle and IFT value related to the fluid without nanoparticle (100 SW) gives the lowest oil recovery. The results indicate that the main effective mechanism of investigated nanohybrid is wettability alteration toward more water-wetness. Adding nanoparticles to the brine with varied salinity reduces the IFT value; however, this reduction with changing the base fluid salinity is not very significant.

## 4. Conclusions

Since nanoparticles have some unique properties—such as ultra-small size, very high surface-to-volume ratio, low costs, and low environmental impact—they have been considered as potential agents to enhance oil recovery. This study investigated the effectiveness of the novel iron-carbon nanohybrid as EOR agent and their corresponding mechanisms through nanofluid flooding into the oil-wet micromodel. The properties of the nanoparticles were explored using XRD analysis, FTIR experiment, zeta potential, and viscometry. The results show that the base fluid salinity enhancement decreases the absolute value of zeta potential of the nanofluids since the higher salinity of the bulk fluid caused the shrinkage of the double layer. The nanofluid used in this study appears stable despite the low zeta potential value. It is observed that the average viscosity of the nanofluids is a function of nanofluid concentration and base fluid salinity, i.e., nanofluid viscosity increases with the enhancement of base fluid salinity and nanoparticle concentration. To achieve better insight regarding liquid–liquid and solid–liquid interactions in porous media, mechanisms involved during nanofluid flooding, a series of sub-pore tests—including interfacial tension (IFT), contact angle, and oil displacement experiments within porous medium—were carried out. The performance of different concentrations of nanoparticles dispersed in a water base fluid with different salinities was evaluated by conducting heavy oil recovery experiments in a glass micromodel. Adding nanoparticles to the brine with different salinities improved the amount of oil recovery from 30% (test SW injection) to a value of 44.3% (test SW + 0.5% injection). As was clearly observed, the solution of 2SW + 0.5% with the lowest contact angle value provides the highest oil recovery (46.2%). However, nanofluid concentration enhancement from 0.05% to 0.5% produce oil only three percent more. In contrast, the highest contact angle value related to the fluid without nanoparticle (100 SW) gives the lowest oil recovery (about 27%). The contact angle measurements have also shown that the significant effect of nanofluid on wettability alteration from the oil-wet surfaces to water-wet surfaces. Therefore, the effective mechanism on oil displacement using an iron-carbon nanohybrid is attributed to the wettability alteration. However, micro-dispersion formation has also contributed to the oil displacement. The current work presents the influence of a novel nanofluid and its relevant mechanisms within strongly oil-wet micromodel. It is recommended that the displacement experiments are repeated within oil-wet core samples to evaluate the effectiveness of the investigated nanoparticles on core-flooding.

## Figures and Tables

**Table 1:** Detailed analysis of SARA and physical properties of heavy crude oil at ambient condition.

Crude Name	$API^{{}^{\circ}}$	Saturates (%wt)	Aromatics (%wt)	Resins (%wt)	Asphaltenes (%wt)	TAN (mg KOH/g)	TBN (mg KOH/g)	Density (gr/cc)	Viscosity (cp)
BG	19.2	51	31	8.3	9.7	0.09	2.94	0.939	251.39

**Table 2:** Salt compositions and TDS of three types of brine solutions used for dynamic oil displacement, IFT, and contact angle tests.

Ion Compositions	SW (mglit^−1^)	2 SW (mglit^−1^)	100 W (mglit^−1^)
Na^+^	12,355	6177.5	123.5
Cl^−^	21,981	10,990.5	219.81
Ca^2+^	440	220	4.4
Mg^2+^	1360	680	13.60
K^+^	375	187.5	3.75
SO_4_^2−^	2740	1370	27.4
HCO^3−^	200	100	2
Sr^2+^	7.5	3.75	0.075
Fe^2+^	1	0.5	0.01
Al^3+^	<1	<0.5	<0.01
Li^+^	<1	<0.5	<0.01
TDS (ppm)	39,182	19,591	391.82

**Table 3:** Physical and petrophysical characteristics of the micromodels.

Dimension (cm)	Pore Diameter (µm)	Etched Depth (µm)	Pore Volume (mL)	Porosity (%)	Absolute Permeability (D)
3.5 × 3.5	16–270	166	0.08	39	1.1

**Table 4:** List of oil displacement experiments in the present study.

Test No.	Injected Nanofluid (Base Fluid + Nanoparticle Concentration wt %)	Flow Rate $\left(\mathrm{mL}\cdot \min^{-1}\right)$	Capillary Number
1	SW + 0.05%	0.00108	1 × 10^−6^
2	SW + 0.5%	0.0017	1 × 10^−6^
3	2SW + 0.05%	0.00106	1 × 10^−6^
4	2SW + 0.5%	0.0017	1 × 10^−6^
5	100SW + 0.05%	0.0018	1 × 10^−6^
6	100SW + 0.5%	0.0015	1 × 10^−6^

## Data Availability

The data presented in this study are available on request from the corresponding author.
